# Effects of Stress, Vagal Nerve Stimulation and Disease Activity on Circulating Cytokines, Quantified by an Ultrasensitive Technique, in Ulcerative Colitis: A Pilot Study

**DOI:** 10.1002/jgh3.70206

**Published:** 2025-06-28

**Authors:** Tamara Mogilevski, Melinda Y. Hardy, Kazuya Takahashi, Rebecca Smith, Anke L. Nguyen, Adam Farmer, James O. Lindsay, Jason A. Tye‐Din, Qasim Aziz, Peter R. Gibson

**Affiliations:** ^1^ Department of Gastroenterology School of Translational Medicine, Monash University and Alfred Health Melbourne Australia; ^2^ The Royal London Hospital, Barts Health NHS Trust London UK; ^3^ Centre for Neuroscience, Surgery and Trauma Blizard Institute, Wingate Institute of Neurogastroenterology, Barts and the London School of Medicine and Dentistry, Queen Mary University of London London UK; ^4^ Immunology Division Walter and Eliza Hall Institute Melbourne Australia; ^5^ Department of Gastroenterology and Hepatology St Louis University St Louis USA; ^6^ Department of Gastroenterology The Royal Melbourne Hospital Melbourne Australia

**Keywords:** basic science, biomarkers, clinical trials, experimental models and pathophysiology

## Abstract

**Aims:**

The utility of circulating cytokine concentrations in ulcerative colitis is limited due to poor assay sensitivity. We aimed to examine the relationship of circulating cytokine concentrations, when measured by ultrasensitive detection, to disease activity, response to induction therapy, and physiological stimuli associated with mucosal injurious events.

**Methods:**

Plasma was obtained from adult patients with ulcerative colitis in whom disease activity was assessed; before/after induction therapy; and before/after acute psychological stress and cervical transcutaneous vagal nerve/sham stimulation. Cytokines were measured using an ultrasensitive electrochemiluminescence‐based multiplex assay. For stress/vagal‐stimulation studies, epithelial injury was quantified by plasma concentrations of intestinal‐type fatty acid‐binding protein.

**Results:**

In 12 patients in remission, 15 with mild‐moderately active disease and 6 with severe colitis, concentrations of interleukin‐17, IL10, interferon‐**γ**, interleukin‐8 and interleukin‐6, but not tumor necrosis factor‐α or interleukin‐12p70, significantly reflected disease activity. In five patients in whom clinical remission was achieved with induction therapy, only interleukin‐12p70 changed with a median increase of 65 (IQR 42–303)% (*p* = 0.006), with no consistent changes in the 10 not in remission. Vagal nerve stimulation had no effect on cytokine concentrations, but, following stress, interleukin‐12p70 increased by 37 (13–60)% compared with a reduction of 29 (3–55)% following sham stimulation (*p* = 0.012), an effect that mirrored that of epithelial injury.

**Conclusion:**

Multiple cytokines were detected at sub‐pg/mL levels and many showed relationships to disease activity in patients with ulcerative colitis. Effects exerted by interventions associated with epithelial injury were consistently detected by changes in interleukin‐12p70 concentrations, but not other cytokines.

**Trial Registration:** Australian New Zealand Clinical Trials Registry: ACTRN12621000168853 and ANZCTR 12620000569909 [Ultrasound studies]; ClinicalTrials.gov identifier: NCT03908073

## 1 Introduction

Clinical application of circulating cytokine levels as a surrogate “window” to intestinal mucosal injury and inflammation has suffered from the lack of sufficient sensitivity of traditional assays to accurately quantify the very low levels. Advances in technology have enabled ultra‐sensitive cytokine detection in the sub‐pg/mL range with multiplex functionality using approaches such as the electrochemiluminescence‐based Mesoscale Discovery assay (MSD). By sensitively detecting antigen‐specific immune responses, MSD assays have shown potential clinical utility in the assessment of a broad range of immune‐associated diseases including infections, such as COVID‐19 [1], autoimmunity [2] and cancers [3]. In Crohn's disease, six biomarkers detected by MSD showed better prediction of IBD disease activity than routine measures such as CRP and fecal calprotectin [4] and, in another study, the serum concentrations of IL12/23p40 and IL12p70 significantly increased in patients achieving endoscopic remission [5]. There has been limited application of these assays in patients with ulcerative colitis and in situations where more subtle changes, especially in patients without detectable disease activity, might be anticipated.

The current study aimed to explore two questions. First, do levels of circulating cytokines detected by the ultra‐sensitive MSD assay system reflect variations of intestinal inflammation in patients with ulcerative colitis? To address this, a cross‐section of patients was studied together with a sub‐group who were evaluated before and after induction medical therapy. Second, do the effects on the intestinal mucosa of acute stress, with and without transcutaneous vagal nerve stimulation, manifest as changes in intestinal barrier function or circulating cytokine levels? To address this, a double‐blind, placebo‐controlled, cross‐over study in patients who had ulcerative colitis in remission was undertaken. A validated stress‐induction paradigm [6] and transcutaneous vagal nerve stimulation, hypothesized to have systemic and local intestinal anti‐inflammatory and intestinal barrier‐promoting effects [7, 8], were applied. The acute effects of these interventions on concentrations of intestinal fatty acid‐binding protein (IFABP), as a biomarker of small intestinal epithelial injury [9] and of a panel of cytokines, were evaluated.

## 2 Materials and Methods

### 2.1 Participants

Plasma samples were obtained between July 2018 and January 2021 from patients of the IBD Clinics at the Alfred Health and Monash Health, Melbourne, who had been recruited into separate clinical study protocols [10, 11]. Patients with ulcerative colitis who were commencing a new medical therapy and had active disease, defined clinically as having a pMayo score > 1, sonographically active disease (bowel wall thickness > 3.0 mm in the diseased bowel segment/s) or endoscopically active disease (defined as Mayo endoscopy subscore > 1), and patients admitted for intravenous corticosteroids due to severe ulcerative colitis were studied. All cohorts were 18 years of age or over, had no evidence of perianal disease, and no superimposed infection.

Patients for the vagal nerve stimulation study were recruited from the Barts Health IBD Clinic, London, between November 2018 and March 2020. Suitability of study entry was assessed by the treating clinician and confirmed by the study gastroenterologist. Patients between 18 and 76 years of age, and with a diagnosis of ulcerative colitis at least 3 months before were included. Patients needed to have a pMayo score < 4 within 3 months of study entry and a fecal calprotectin < 250 μg/g within 2 weeks of study entry, be on a stable medication regimen for the prior 3 months. Exclusion criteria included the presence of a stoma; currently taking anti‐TNF therapy, thiopurines, methotrexate, topical or oral corticosteroids; pregnancy or lactation; known or suspected cerebrovascular disease; clinically significant abnormal screening electrocardiogram, known or suspected cardiac disease or poorly controlled hypertension; previous cervical vagotomy, an implanted electrical and/or neurostimulator device or previous use of the gammaCore device or history of syncope or seizures (within last 2 years).

### 2.2 Protocol for the Non‐Interventional Arm

At study entry, baseline demographics, clinical activity via the pMayo score and serum C‐reactive protein (CRP) were recorded. At the follow‐up visit, disease activity measures were documented. Response was defined strictly as pMayo 0 on follow up. For in‐patients with severe colitis, patient care was managed by the treating clinical team who were independent of the study investigators.

### 2.3 Vagal Nerve Stimulation and Stress Protocol

**FIGURE 1:**
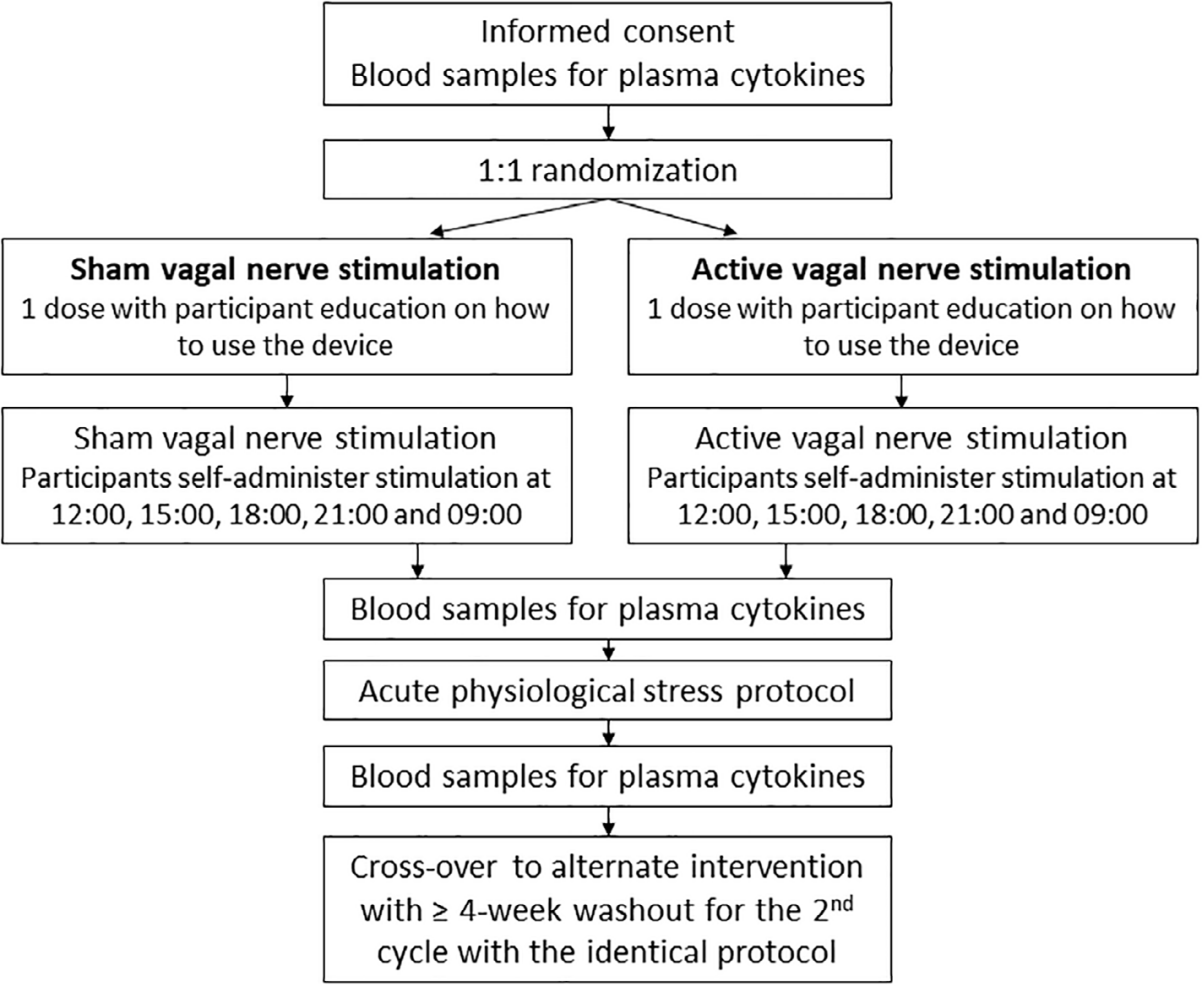
Protocol followed in patients with ulcerative colitis in remission (fecal calprotectin < 50 μg/g) who had vagal nerve and sham stimulation and were subjected to acute psychological stress, alone or in combination. Endpoints were cytokine concentrations in plasma.

A flow chart of study procedures can be found in Figure 1. The interventions were carried out within two cycles, each comprising two study visits. Patients were screened for eligibility by a gastroenterologist. They presented for Visit 1 between 08:00 and 09:00, 20 mL of venous blood was drawn for later analysis, and underwent a screening electrocardiogram. Eligible patients were randomized, using www.randomisation.com, to receive either active or sham transcutaneous vagal nerve stimulation. In order to maintain double blinding, a member external to the project selected and administered the active or sham vagal stimulation. Patients were administered a single 2‐min bilateral stimulation (with left followed by right cervical vagal nerve stimulation) and were subsequently instructed on how to use the device independently. Details of the vagal stimulation methodology are outlined in Data S1. Patients and the study clinician were blinded to the intervention that they received.

The patients then returned to their daily activities where they self‐administered five further 2‐min bilateral stimulations at 3‐h intervals while awake. The following day they returned to the laboratory where they self‐administered the last dose of vagal or sham stimulation, after which time a further 20 mL of venous blood was taken for analysis. The participants were then subjected to the stress protocol, as previously reported [6] (see details in Data S1). Further blood samples were taken after 45 min of rest. This concluded Cycle 1. Patients were then invited to return after a period of no less than 4 weeks for two further study visits—Cycle 2—with an identical protocol, but receiving the alternate intervention.

### 2.4 Biomarker Analyses

Extracted plasma from the venous blood samples were stored in aliquots at −80°C until assayed. A U‐PLEX 9‐plex assay from Meso Scale Diagnostics LLC (Rockville, MD, USA) was performed according to manufacturer's instructions. The 9‐plex panel assessed interferon‐γ, interleukin (IL)‐1β, IL‐6, IL‐8, IL‐10, IL‐12p70, IL‐13, IL‐17a and tumor necrosis factor‐α (TNFα), chosen on the basis of prior results from studies involving vagal nerve stimulation and stress responses in patients with ulcerative colitis [6, 12]. Cytokine concentrations were determined using the MSD QuickPlex SQ 120MM plate reader and Discovery Workbench 4.0 software. The lower limit of quantification (LLOQ) was calculated for each cytokine on each assay plate based on the standard curve. Calculated values below the LLOQ were reported as equal to the LLOQ. For the interventional studies in patients with ulcerative colitis, concentrations of IFABP, were measured by ELISA (human IFABP, R&D Systems, USA), according to manufacturer's instructions.

### 2.5 Ethical Considerations

Protocols for the studies were approved by the Alfred and Monash Health Office of Ethics and Research Governance and the London Surrey Borders Research Ethics Committee (approval number 18/LO/18). The ultrasound studies were registered on the Australian New Zealand Clinical Trials Registry (ACTRN12621000168853, and ANZCTR12620000569909) and the vagal nerve stimulation study on Clinicaltrials.gov (identifier NCT03908073).

### 2.6 Statistical Analysis

Data are presented as median with interquartile range (IQR) differences between the biomarkers and cytokines at different time points were evaluated using the Wilcoxon signed‐rank test or paired *t*‐test and differences between levels of cytokines across patients in remission, with active disease and with acute severe colitis were calculated using the Mann–Whitney *U* test. The level of statistical significance was set at *p* ≤ 0.05. All statistical analyses were performed using Prism V8.30 (GraphPad Software LLC).

## 3 Results

### 3.1 Patients

Thirty‐three patients, 12 with ulcerative colitis with quiescent disease, 15 with mild‐moderately active and six with severe disease, were studied. Their demographic details, and clinical and laboratory features are shown in Table 1. All patients recruited into the quiescent arm had fecal calprotectin measurements < 50 μg/g. Of the 12 patients randomized for the vagal nerve stimulation study, 11 completed the full four visits, with one withdrawing after one study arm due to hesitation about blood collection. There were no adverse events reported in relation to vagal or sham stimulation.

**TABLE 1:** Baseline demographics of patients with ulcerative colitis. Results are shown as number (%) or median (range).

		Disease activity		
		Remission	Mild–moderate	Severe
Number		12	15	6
Age, years		43 (19–72)	33 (28–54)	32 (25–34)
Female sex (% total)		9 (67%)	6 (40%)	1 (17%)
Disease extent	E1	3 (25%)	4 (27%)	0 (0%)
	E2	5 (42%)	9 (60%)	4 (67%)
	E3	4 (33%)	2 (33%)	2 (33%)
Medications	Mesalazine	Oral 9; rectal 1; both 1		Oral [3]
	Thiopurine		1	2
	TNF inhibitor		Infliximab 3; adalimumab 2	Infliximab 1
	Vedolizumab		8	
	Ustekinumab		1	
Clinical indices	pMayo	0 (0–0)	5.5 (1.5–7)	9 (8–9)
	Fecal calprotectin, μg/g	24 (< 12.5–79)	765 (76–7977)	1653 (1414–1934)
	C‐reactive protein, mg/L	1 (< 1–4)	3.5 (1–9)	50 (30–115)

### 3.2 The Effect of Disease Activity on Cytokine Levels

**FIGURE 2:**
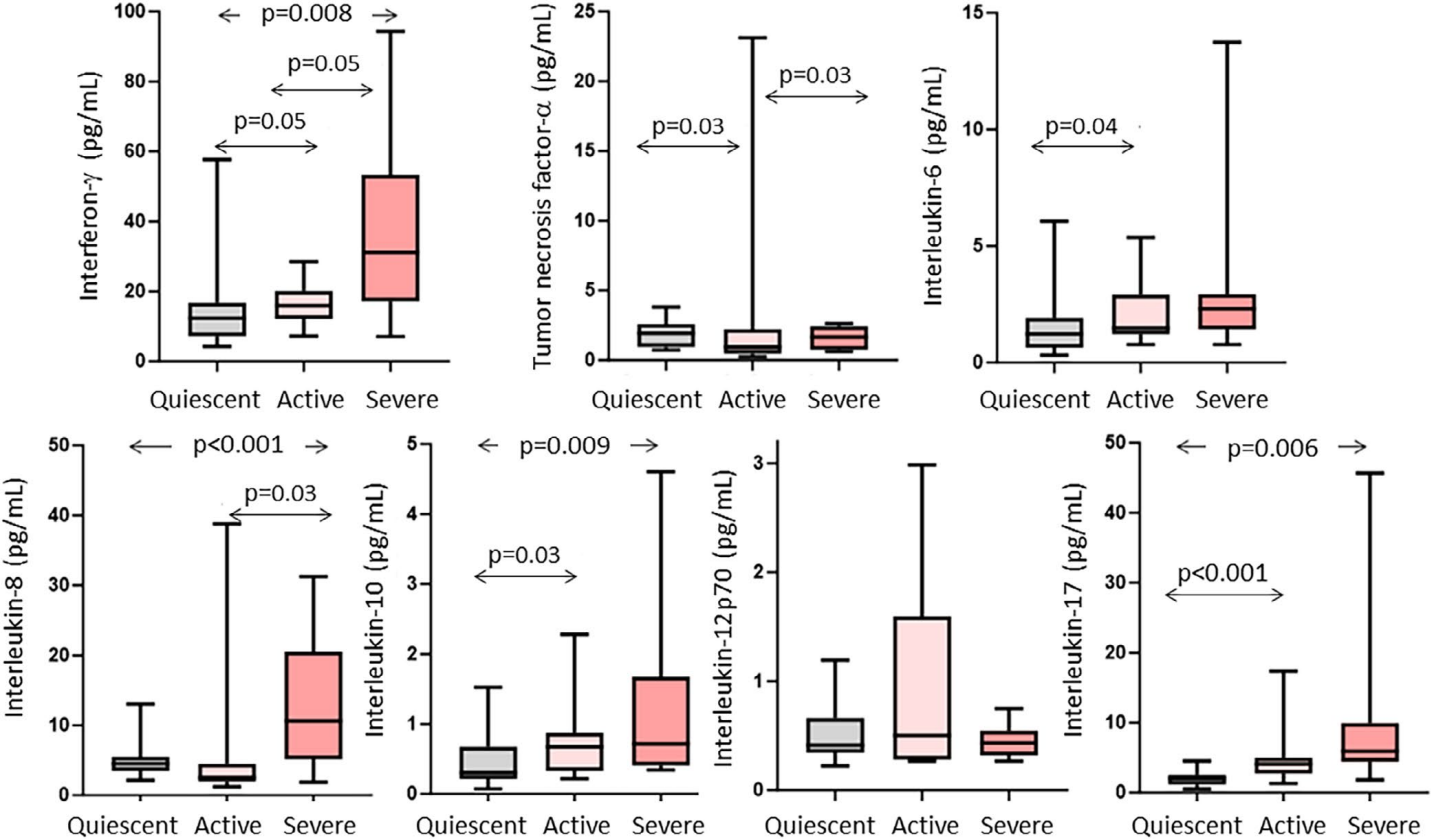
Cytokine levels in plasma from patients with ulcerative colitis according to disease activity. Data shown as box‐plots. Statistical comparisons performed using the Mann–Whitney *U* test.

IL13 and IL1β could not be measured in half of the participants with disease in remission or active disease. The results of the remaining seven cytokines were above detection limits for the vast majority of the participants. Four patterns were evident (Figure 2). First, interferon‐γ, IL10, IL17 and IL8 increased with increasing disease activity. Second, IL6 was increased between the remission and active group, but did not increase further in those with severe disease. Third, TNFα decreased in the active compared to remission and severe groups. Lastly, IL12p70 showed no activity‐related differences.

**FIGURE 3:**
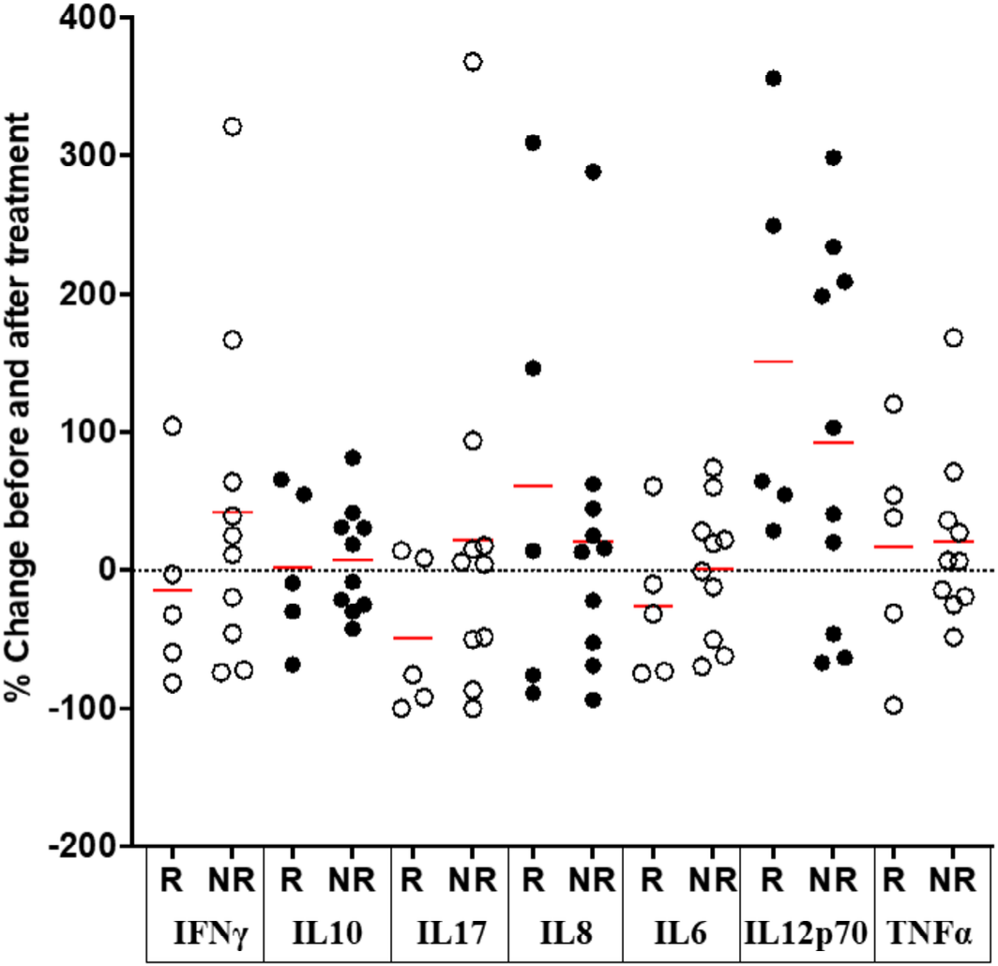
Changes in circulating cytokine concentrations of patients with active ulcerative colitis after 14–19 weeks' change in therapeutic agent according to whether complete clinical remission (pMayo = 0) was achieved (R) or not (NR).

Cytokine concentrations were measured in the cohort with mild‐moderately active disease (*n* = 15) before and 14–19 weeks after the initiation of a new treatment (Table 1). In the five patients who went into clinical remission, from a median pMayo score of 6 (range 0–8) to 0, the only cytokine showing consistent changes was IL12p70, for which the concentrations increased from median 0.2 (IQR 0.05–0.7) to 0.4 (0.3–1.0) pg/mL, representing an increase of 65 (42–303)% (*p* = 0.006; paired *t*‐test) (Figure 3). In the other 10 patients, the pMayo score changed from 4 [2, 3, 4, 5, 6] to 2 [1, 2, 3, 4, 5] and no consistent differences were observed in cytokine levels between the two time periods (Figure 3).

### 3.3 Effect of Transcutaneous Vagal Nerve Stimulation on Cytokines in an Unstressed State

**FIGURE 4:**
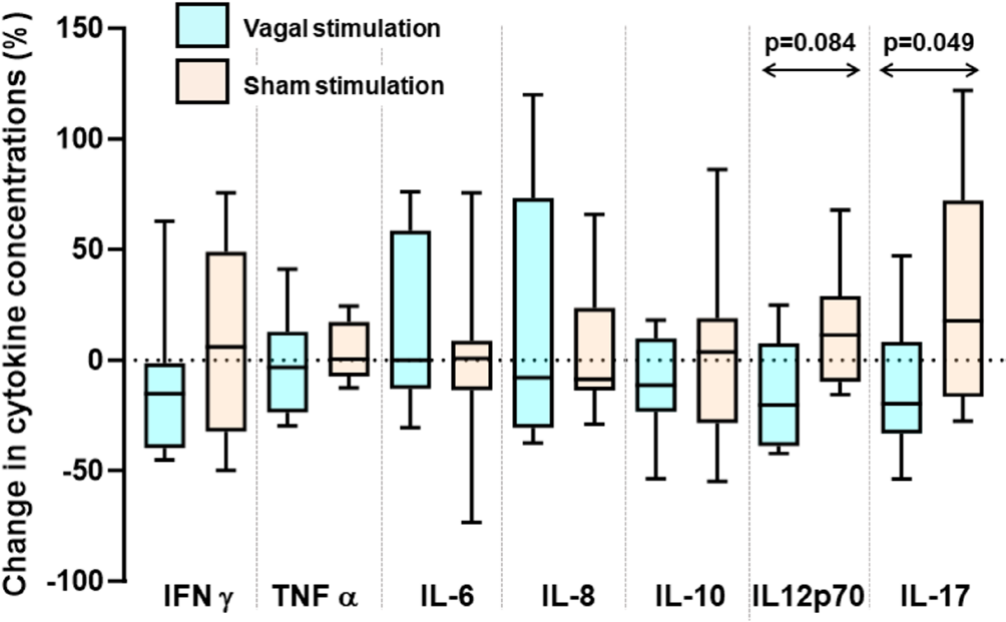
Box‐plots of changes in the concentrations of cytokines prior to and following 24‐h bilateral vagal nerve or sham stimulation in 11 patients with ulcerative colitis who had paired samples. Two patients had interferon‐γ levels in single samples in the sham arm that were outliers (> 3 SD of other results) and are not shown in the graph, but included in the analysis. Statistical comparisons between paired data within and between each intervention were performed using the Wilcoxon signed‐rank test. All *p*‐values were > 0.10 except those shown in the graph.

**FIGURE 5:**
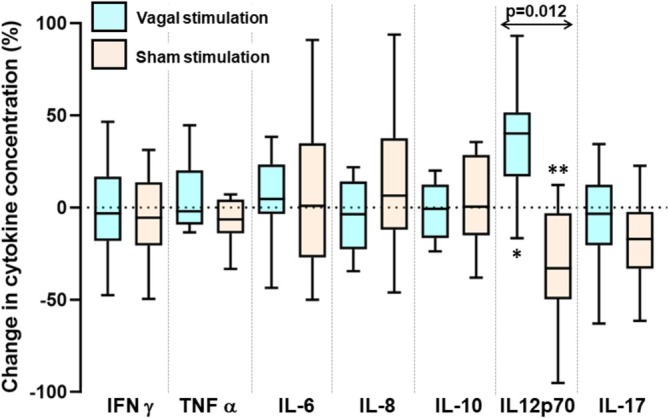
Box‐plots of changes in the concentrations of cytokines prior to and following undergoing the stress paradigm in 11 patients with ulcerative colitis who had undergone 24‐h bilateral vagal nerve or sham stimulation. For IL12p70, data from one patient was below the detection limit, but included in the non‐parametric analysis. Concentrations of IL 12p70 reduced with stress after sham stimulation (**p = 0.039; Wilcoxon signed‐rank test) and tended to increase with stress after vagal nerve stimulation (*p = 0.055). The stress‐induced changes after vagal nerve stimulation differed from those after sham stimulation (*p* = 0.012). No statistically significant differences were observed for all other cytokines (all *p* > 0.1).

After vagal stimulation, IFAB concentrations were 1351 (1008–1693) pg/mL compared with 1504 (1142–1865) pg/mL after sham stimulation (*p* = 0.06; paired *t*‐test). Cytokine levels following 24‐h vagal stimulation and the change from pre‐stimulation levels were similar to those following sham stimulation for all cytokines, with two exceptions (Figure 4). For IL‐17, neither vagal nerve nor sham stimulation significantly changed concentrations (*p* > 0.05 for both), but the reduction by 20 (−8 to 33)% of concentrations with vagal nerve stimulation was different from the increase of 18 (−17–72)% with sham stimulation (*p* = 0.049; Figure 5). For IL12p70, concentrations were 0.48 (0.32–0.76) pg/mL before and 0.37 (0.24–0.64) pg/mL after vagal stimulation (*p* = 0.13) compared with 0.39 (0.34–0.56) pg/mL before to 0.46 (0.44–0.67) pg/mL (*p* = 0.28) after sham stimulation. Paired analysis of changes associated with vagal nerve stimulation with those from sham stimulation showed a trend towards significance (*p* = 0.084; Figure 4).

### 3.4 Effect of Vagal Nerve Stimulation on Biomarkers in a Stressed State

The stress protocol induced anxiety (see Data S1). After stress, concentrations of IFABP were 1265 (784–1746) pg/mL with vagal stimulation compared with 1886 (811–2961) pg/mL with sham stimulation (*p* = 0.05; paired *t*‐test; Figure S2). Compared with levels prior to stress, this represented a reduction of 11 (−2 to 24)% after vagal stimulation compared with an increase of 16 (−15.1–48)% with sham stimulation (*p* = 0.02; Figure S2).

The stress protocol after transcutaneous vagal or sham stimulation was associated with changes in the concentrations of IL12p70, but not the other cytokines (Figure 5). Vagal nerve stimulation tended to reduce stress‐induced IL12p70 levels from 0.46 (0.39–0.67) to 0.37 (0.24–0.64) pg/mL (*p* = 0.055, Wilcoxon ranked‐sign test), compared 0.31 (0.21–0.46) to 0.46 (0.39–0.67) pg/mL with sham stimulation (*p* = 0.039). In paired analysis of changes induced, the concentrations of IL12p70 in response to stress increased with vagal stimulation by 37 (13–60)% in contrast to a decrease of 29 (3–55)% with sham stimulation (*p* = 0.012), representing an overall effect size of 66%. Given the crossover design of the study, all results were analyzed by order of intervention with no impact on the above findings detected.

## 4 Discussion

Application of a new ultrasensitive technique of quantifying cytokines in plasma to cohorts of patients with ulcerative colitis showed all cytokines examined, except IL‐13 and IL1β, were readily measurable within the linear concentration range in most samples. An approximately linear relationship was observed between increasing disease activity, when classified via the pMayo score as remission, active and severely active, and concentrations of IL17, IL10, interferon‐γ, IL8 and IL6, while a bell‐shaped curve for TNFα and no associations with IL12p70 were detected. However, inflammatory response to induction therapy and interventions that altered mucosal injury and/or inflammation was consistently reflected only in concentrations of IL12p70.

That disease activity correlated with circulating concentrations of IL17, IL10, IL8, IL6 and interferon‐**γ** when assessed in cross‐sections of patients with ulcerative colitis is not new [4, 13, 14, 15], as was the poor correlation with levels of TNFα [15, 16]. Despite this, changes with treatment‐induced improvement in disease activity was not reflected the concentrations of those cytokine levels. In contrast, plasma concentrations of IL12p70 showed no overall relationship with disease activity, but its levels increased in all five patients who achieved complete remission (as shown by pMayo 0) following treatment. This finding is consistent with observation of increased circulating IL12p70 in patients with Crohn's disease who achieve endoscopic remission [5]. Since that study also employed MSD technology, it raises the possibility that ultrasensitive detection is necessary to detect a meaningful signal, which may explain the novelty of our observation. A possible explanation for the increase in IL12p70 with remission may be reactivation of TH1 immunity as inflammation decreases, which may represent a homeostatic mechanism; elevated IL12p70 may drive a healthy immune response capable of responding to infections or other challenges, while mucosal healing is underway.

Given the considerable overlap of cytokine levels across the degrees of inflammation and the generally non‐informative changes in association with induction therapy, their clinical utility as markers of inflammation is dubious, without deeper analysis of well‐matched populations. While the patients examined before and after induction therapy were not assessed endoscopically or via fecal calprotectin at the end of therapy, the correlation of symptoms and inflammation is reasonable in patients with ulcerative colitis [17]. Furthermore, the cohort in remission had been so with normal fecal calprotectin levels for at least 3 months, suggesting a deeper level of healing than those responding to induction therapy. The clinical utility of IL12p70 as a biomarker of response to treatment in patients with ulcerative colitis would require endoscopic and histological data paired with plasma cytokine levels early in the course of disease treatment.

As a research tool, however, the application of ultrasensitive detection techniques might have impact. There is a need for methods to quantify acute mucosal changes after exposure to conditions that potentially injure or inflame the intestine. Stress and vagal nerve stimulation were chosen as the altered “physiological” conditions to evaluate this concept. Stress imparted by the dichotomous listening protocol had been previously shown to induce rectal mucosal inflammatory and vascular changes [6]. In order to exaggerate effects of the stress, it was combined with vagal nerve stimulation, which has anti‐inflammatory effects in studies of experimental animals and healthy human volunteers [7], and suppresses stress‐induced increase in small intestinal permeability in healthy adults [18]. In order to determine if such manipulations were having any effects on mucosal homeostasis, the circulating concentrations of IFABP, a marker of small intestinal epithelial injury [9], were concomitantly measured. There was a 10% reduction of IFABP levels compared with those with sham stimulation for vagal nerve stimulation in an unstressed state. This was exaggerated when applied in stressed participants, suggesting that transcutaneous vagal nerve stimulation was protective against the negative effects of stressful stimuli on small intestinal epithelial injury.

The cytokine responses to stress and vagal nerve stimulation were unremarkable for most cytokines. We were unable to show changes in the circulating concentrations of IL10, IL6, and TNFα after stress, as previously reported in health volunteers [12, 18, 19], and for IL6 in patients with post‐traumatic stress disorder undergoing stress [20]. This discrepancy may have related to the different methodology used for their measurement and the relatively small numbers of patients studied. The reduction in circulating IL17 observed with stress in the current study has not been previously reported and is at odds with the current understanding of stress causing an inflammatory reaction via the IL23/IL17 pathway [21]. However, vagal stimulation with or without stress had no significant effect on IL17 levels.

The most notable observations were the consistent changes in the concentrations of IL12p70. In the unstressed patient, vagal nerve stimulation tended to be associated with lower concentrations of IL12p70 compared with those after sham stimulation, effects that mirrored those on IFABP. Stress‐induced suppression of IL12p70 concentrations was reversed by vagal stimulation and levels were 66% greater than those following sham stimulation with stress, again mirroring the effects on IFABP levels. Existing evidence suggests that secretion of IL12 is impaired with stress both in vitro [22] and in animals in vivo [23]. Both models, as in the present study, measured the IL12p70 heterodimer, which is specific to IL12 activity [24]. It is hypothesized that, through this mechanism, stress can have a negative impact on T helper 1 cellular responses and tumor immunosurveillance [25]. This finding requires replication in larger cohorts prior to drawing conclusions from this result.

The study has limitations. First, the small number of patients in each group was anticipated given its pilot study design, but the results are important to transmit as this ultrasensitive assay system is expensive in its hardware and the reagent kits. Second, therapy was heterogeneous across the groups with, for example, biologic therapy in none of the remission group, in 15 of 16 active group and three in the severe group), which may have influenced cytokine expression independently of inflammatory activity. However, despite this limitation, a cohort well characterized according to disease activity criteria with objective confirmation of presence or absence of inflammation, were studied with relevant results obtained to guide future prospective research in larger and specified cohorts of patients. The results found should enable the generalizability of the findings to be determined. Third, the range of cytokines could be expanded to include other critical components of the IL12/IL‐23 pathway, such as IL‐12p40 that is available and IL‐23p19 that is currently not available of the MSD platform. Finally, the retrospective nature of this study did not enable detailed objective assessment of the inflammatory status of the patients in the ‘active’ group and, hence, the relationship to severity could only be broadly assessed. Future studies addressing specific hypotheses that arise from the finding of the current pilot study are warranted.

In conclusion, measurement of a panel of cytokines in the peripheral blood using ultrasensitive MSD assays can be quantitatively performed in patients with ulcerative colitis. Even though several inflammatory cytokines may act as a guide to gross differences to the degree of mucosal colonic inflammation, changes in their circulating concentrations did not reflect improvement in inflammatory status with induction therapy. They are also not informative of acute changes in mucosal effects of stress with or without vagal nerve stimulation in patients in remission with a normal fecal calprotectin. In contrast, the circulating level of IL12p70, a cytokine with no consistent relationship to the degree of inflammation, consistently reflected subtle changes induced by the acute interventions (stress and vagal nerve stimulation) that concomitantly were associated with differences in a biomarker of small intestinal injury, and by induction of clinical remission by therapy. Thus, measurement of circulating cytokines using an ultrasensitive technique has potential to provide insight into pathogenic mechanisms in the colonic mucosa in patients with ulcerative colitis in the clinical and research settings.

## Ethics Statement

The studies were approved by the Alfred and Monash Health Office of Ethics and Research Governance and the ultrasound studies were registered on Australian New Zealand Clinical Trials Registry (ACTRN12621000168853, and ANZCTR 12620000569909, respectively). For the studies on vagal nerve stimulation and stress, the protocol was approved by the London Surrey Borders Research Ethics Committee (approval number 18/LO/18).

## Consent

Prior to entry in the studies, patients signed written informed consent.

## Conflicts of Interest

Tamara Mogilevski: Speaker honoraria from for Takeda and Sandoz. Melinda Y. Hardy: consultant for Takeda. Kazuya Takahashi: No conflicts declared. Rebecca Smith: Speaker honoraria received from AbbVie and Johnson & Johnson. Anke L. Nguyen: Speaker honorarium from Dr. Falk Pharma. Adam Farmer: No conflicts declared. James O. Lindsay: has received investigator‐initiated research support from AbbVie, Gilead, Pfizer, Shire and Takeda; Honoraria for consultancy of speaking from AbbVie, BMS, Celgene, Celtrion, Engytix, Ferring, Galapagos, Gilead, GSK, Janssen, Lilly, MSD, Pfizer, Shire, Takeda. Jason A. Tye‐Din: consultant or advisory board member for Anatara, Anokion, Barinthus Biotherapeutics, Chugai Pharmaceuticals, DBV Technologies, Dr. Falk, Forte Biosciences, IM Therapeutics, Janssen, Kallyope, Mozart Therapeutics, TEVA and Topas, has received research funding from Barinthus Biotherapeutics, Chugai Pharmaceuticals, Codexis, DBV Technologies, Kallyope, Novoviah Pharmaceuticals, Topas and Tillotts Pharmaceuticals and received Honoraria from Takeda. He is an inventor on patents relating to the use of gluten peptides in coeliac disease diagnosis and treatment. Qasim Aziz: Principal Investigator in clinic trials for Takeda, Classado Pharma and Dr. Falk Pharma. Peter R. Gibson: consultant or advisory board member for Anatara, Atmo Biosciences, Topas and Comvita; research grants for investigator‐driven studies from Mindset Health, and speaker honoraria from Dr. Falk Pharma and Mindset Health Pty Ltd. Shareholder in Atmo Biosciences.

## Supporting information


**Data S1.** Supporting Information.

## Data Availability

The data underlying this article will be shared on reasonable request to the corresponding author.
