# Supplementary material for: Effects of Stress, Vagal Nerve Stimulation and Disease Activity on Circulating Cytokines, Quantified by an Ultrasensitive Technique, in Ulcerative Colitis: A Pilot Study
Source: JGH Open. 2025 Jun 28;9(7):e70206. doi: 10.1002/jgh3.70206 (PMC12206241; doi:10.1002/jgh3.70206)

**SUPPLEMENTARY INFORMATION**

**Figure s1.** Change in self-reported anxiety levels as shown by a 10-point Likert scale associated with the stress paradigm when applied twice (cycle 1 and cycle 2) to patients with ulcerative colitis in remission. Changes from baseline to timepoint value were all statistically significant (p≤0.02; Wilcoxon test).


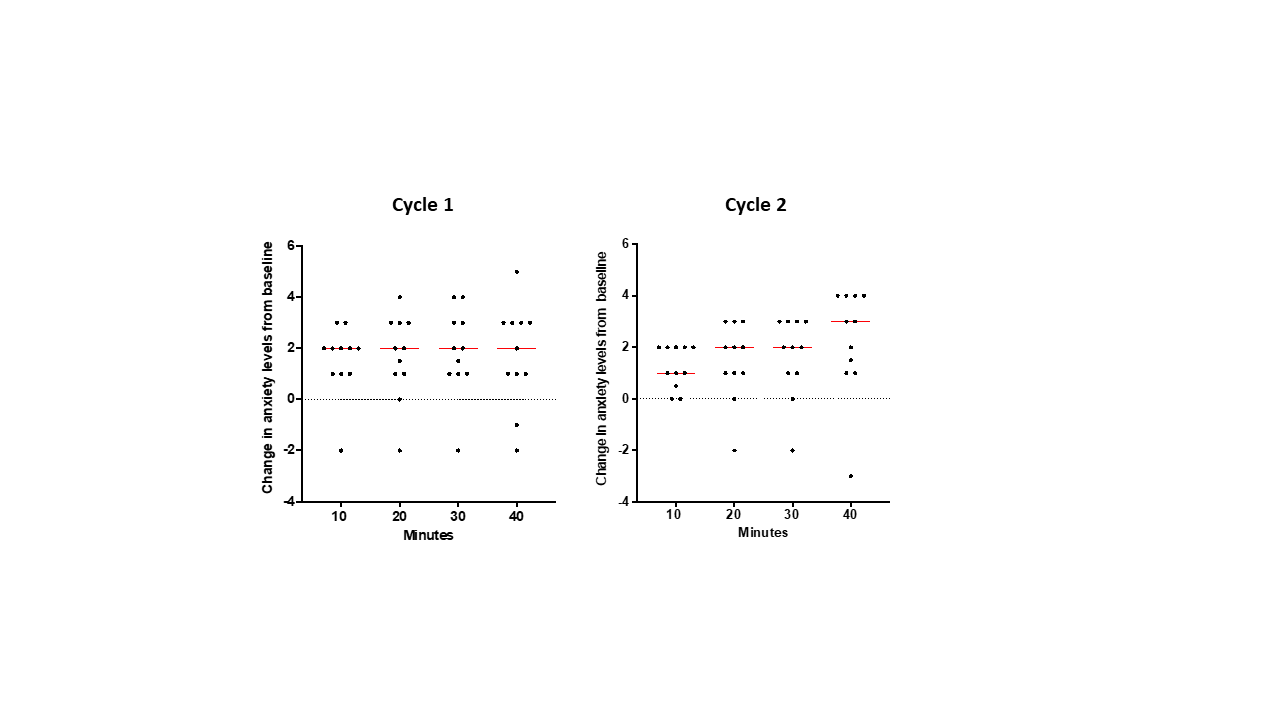


**Figure s2.** The effect of stress and vagal stimulation on plasma concentrations of intestinal fatty acid-binding protein (IFABP) and of its change from pre-stress levels. Horizontal red lines represent mean values. Analyses performed using paired t-test.


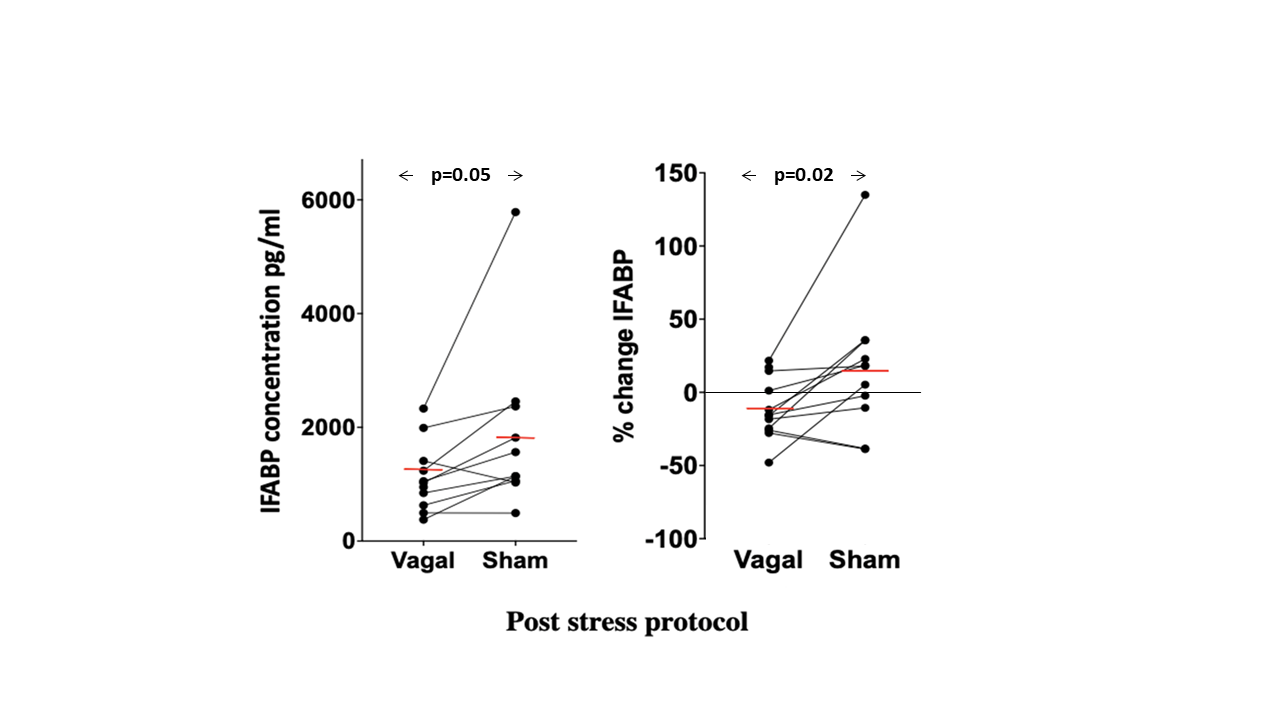

Supplement: Supplementary file 1 — Data S1. Supporting Information. [file JGH3-9-e70206-s001.docx]
